# Narcolepsy Type 2 in an Adolescent With Childhood Obstructive Sleep Apnea and Coexisting Absence Epilepsy: A Case Report

**DOI:** 10.7759/cureus.107464

**Published:** 2026-04-21

**Authors:** Gaurav Sahu, Vinod Mamraj Saini, Shreeja Nair

**Affiliations:** 1 Department of Respiratory Medicine, Mahatma Gandhi Mission (MGM) Medical College and Hospital, MGM Institute of Health Sciences, Navi Mumbai, IND

**Keywords:** adolescents, excessive daytime sleepiness, hypnopompic hallucinations, multiple sleep latency test, narcolepsy type 2, periodic limb movements, polysomnography, sleep paralysis

## Abstract

Narcolepsy is a chronic neurological disorder of sleep-wake regulation whose cardinal features include pathological daytime somnolence and dysregulation of rapid eye movement (REM) sleep. It is frequently underdiagnosed in adolescents owing to symptom overlap with psychiatric, behavioral, and prior sleep disorders, including childhood obstructive sleep apnea (OSA). Narcolepsy type 2 (NT2), defined by excessive daytime sleepiness (EDS) without cataplexy, poses particular diagnostic challenges due to the absence of a single pathognomonic feature. We report an 18-year-old female who presented with a four-year history of persistent EDS, hypnopompic hallucinations, sleep paralysis, and automatic behaviors during microsleeps, including episodes of irrelevant writing during examinations. Subjective daytime somnolence was severe, with an Epworth Sleepiness Scale (ESS) score of 19/24. Past history was notable for childhood OSA managed with adenoidectomy, with residual mild snoring. Physical and neurological examinations were unremarkable. Polysomnography (PSG) demonstrated markedly short sleep latency, multiple sleep-onset REM periods (SOREMPs), and periodic limb movements without apneic episodes or nocturnal desaturation. The Multiple Sleep Latency Test (MSLT) confirmed severe objective hypersomnolence with a mean sleep latency of 1.25 minutes and SOREMPs in three out of four naps. Electroencephalography (EEG) revealed findings consistent with absence seizures, an uncommonly encountered comorbidity in narcolepsy, raising a rare dual diagnosis of NT2 and absence epilepsy. The prior history of childhood OSA and residual snoring initially confounded the clinical picture, underscoring the necessity of objective sleep testing in adolescents with EDS. PSG and MSLT confirmed the diagnosis while excluding insufficient sleep syndrome and untreated OSA. Periodic limb movements observed on PSG may represent a comorbid disorder or an associated feature of narcolepsy. The coexistence of absence epilepsy with NT2 is a rarely reported clinical phenomenon, and its presence in this case further underscores the diagnostic complexity and the critical need for comprehensive neurological evaluation in adolescents presenting with EDS. NT2 in adolescents can be diagnostically challenging due to symptom overlap with prior sleep disorders, psychiatric conditions, and epilepsy. Early recognition, supported by PSG and MSLT, is essential to guide pharmacologic and behavioral interventions, improve daytime function, and reduce psychosocial burden, particularly in rare presentations where narcolepsy coexists with absence epilepsy, demanding heightened clinical vigilance and a broad neurological workup.

## Introduction

Narcolepsy is a chronic neurological disorder of central sleep-wake regulation, fundamentally characterized by excessive daytime sleepiness (EDS) arising from hypothalamic hypocretin (orexin) system dysfunction and the consequent dysregulation of rapid eye movement (REM) sleep architecture [1,2]. The hypocretin system, comprising a small population of lateral hypothalamic neurons that project diffusely across wake-promoting and REM-suppressing circuits, serves as a critical stabilizer of vigilance state boundaries; its compromise, whether through autoimmune-mediated neuronal loss in narcolepsy type 1 (NT1) or through subtler, incompletely characterized mechanisms in narcolepsy type 2 (NT2), engenders the pathological intrusion of REM-sleep phenomena into wakefulness that defines the clinical syndrome [2,3]. Epidemiological data confirm that symptom onset concentrates predominantly within the first two decades of life, with a peak incidence at approximately 15 years of age, establishing narcolepsy unequivocally as a disorder of childhood and adolescence [1,4]. Notwithstanding this well-documented early onset, diagnostic ascertainment in younger patients is protractedly delayed: EDS and ancillary REM-related symptoms, including hypnagogic and hypnopompic hallucinations, sleep paralysis, and automatic behavior, are consistently misattributed to mood dysregulation, behavioral disturbance, insufficient sleep, or intercurrent medical conditions, rather than recognized as manifestations of a primary neurological disorder [5,6].

The diagnostic interval in narcolepsy is among the longest documented for any chronic neurological condition. Multi-center registry data and real-world cohort analyses consistently demonstrate a mean diagnostic latency exceeding one decade from symptom onset to confirmed diagnosis, with adolescent-onset cases disproportionately affected owing to the non-specific nature of EDS in this population and its frequent misclassification as behavioral inattention, affective disorder, or attention-deficit/hyperactivity disorder [7]. Registry data from a Korean multicenter study of 137 patients corroborate this pattern, reporting a mean onset age of 18.2 years against a mean diagnostic age of 28.3 years, a gap of more than 10 years, underscoring persistent and clinically consequential gaps in awareness [7]. In the adolescent, the differential is further confounded by the phenotypic overlap of narcoleptic symptoms with manifestations of psychiatric conditions, circadian rhythm disturbances, and residual sequelae of antecedent sleep-disordered breathing such as obstructive sleep apnea (OSA) [5,6].

Within the narcolepsy spectrum, NT2, defined under the International Classification of Sleep Disorders, Third Edition (ICSD-3) by EDS with objectively confirmed sleep-onset REM periods (SOREMPs) on the Multiple Sleep Latency Test (MSLT), in the absence of cataplexy and with preserved cerebrospinal fluid hypocretin-1 levels, constitutes the most diagnostically challenging subtype [8]. The absence of cataplexy, which in NT1 provides a pathognomonic clinical anchor, renders NT2 a diagnosis of aggregated supportive criteria rather than a single defining feature, substantially increasing the risk of misattribution [8,9]. Long-term observational data have further demonstrated that NT2 may represent a diagnostically unstable entity, with a subset of patients subsequently evolving toward NT1 or alternatively fulfilling criteria for idiopathic hypersomnia, necessitating longitudinal re-evaluation [9].

The published literature on pediatric and adolescent narcolepsy documents a recurrent and clinically consequential pattern of diagnostic error. Gupta et al. reported an adolescent in whom all four cardinal features of the disorder had been sequentially attributed to epilepsy, psychosis, and depression prior to eventual recognition of narcolepsy [4]. The broader impact of this diagnostic failure is substantiated by evidence that pediatric-onset narcolepsy, regardless of subtype, imposes significant burdens on scholastic attainment, neurocognitive function, and psychosocial development [4,6,10]. Of particular relevance is the documented phenotypic overlap between narcoleptic microsleeps with automatic behavior and epileptic absence seizures: Nevsimalova et al. previously described five pediatric patients whose narcoleptic episodes of unresponsiveness were initially misclassified as absence or other seizure types, underscoring the bidirectional diagnostic hazard when these conditions coexist or mimic one another [11]. The coexistence of narcolepsy and epilepsy, while recognized as a rare phenomenon estimated to occur in fewer than 2% of narcolepsy cohorts, represents a clinically distinct and underappreciated diagnostic entity [12,13].

Definitive diagnosis of narcolepsy relies upon formal sleep laboratory evaluation, comprising in-laboratory overnight polysomnography (PSG) to characterize nocturnal sleep architecture and exclude comorbid sleep-disordered breathing, followed on the subsequent morning by the MSLT conducted in accordance with the American Academy of Sleep Medicine (AASM) practice parameters [14]. In the pediatric and adolescent context, accurate interpretation of MSLT findings mandates prior exclusion of insufficient sleep syndrome, circadian rhythm disorders, and untreated OSA, each of which may independently yield spuriously shortened mean sleep latency and artifactual SOREMPs [14]. When paroxysmal episodes of behavioral arrest, unresponsiveness, or stereotyped automatisms are present, supplementary neurophysiological evaluation with electroencephalography (EEG) is essential to delineate ictal from non-ictal events and to identify coexisting seizure disorders, which may both mimic and genuinely coexist with narcolepsy [11,12].

Herein, we present an 18-year-old female with a four-year history of refractory EDS, a constellation of REM-related parasomnic phenomena, and a remote history of childhood OSA, who was ultimately diagnosed with NT2 on objective PSG and MSLT evaluation. The subsequent identification of concurrent absence epilepsy on EEG, a rarely reported dual diagnosis, further stratified the diagnostic complexity of this case, illustrating how epileptic ictal events may phenotypically mimic narcoleptic microsleeps, and vice versa, in the adolescent population. This case reinforces the imperative for systematic, multimodal neurological and polysomnographic assessment, meticulous exclusion of mimicking and comorbid conditions, and heightened clinical vigilance when evaluating adolescents presenting with undifferentiated EDS.

## Case presentation

An 18-year-old girl presented to our sleep clinic with a history of EDS for the past four years. She described an overwhelming urge to sleep that intruded upon ordinary daytime tasks, proving particularly disruptive during classes and examinations, and leading to a decline in scholastic performance. Her parents also noticed that she frequently appeared drowsy during the day, even after seemingly adequate nocturnal sleep. The patient described frequent vivid dreams and excessive dreaming at night, along with episodes of hypnopompic hallucinations on awakening. She also recalled two episodes of transient inability to move on awakening, consistent with sleep paralysis. However, there was no history suggestive of cataplexy, such as sudden muscle weakness triggered by emotions.

During school examinations, she had episodes of irrelevant or incoherent writing, of which she was largely unaware, suggestive of automatic behavior during microsleep episodes. Snoring was present but described as mild in intensity and not associated with witnessed apneas. Her Epworth Sleepiness Scale (ESS) score was 19/24 [15], indicating severe subjective daytime somnolence. A sleep diary maintained over two weeks revealed adequate nocturnal sleep duration with persistent EDS, thus ruling out insufficient sleep syndrome.

Her past history was significant for OSA diagnosed in childhood, for which she underwent adenoidectomy at the age of four years, with reported symptomatic improvement thereafter. There was no recurrence of significant apneic events as per parental history, although occasional snoring persisted. Family history revealed that a first-degree relative had a seizure disorder, raising an initial concern of possible epilepsy.

On physical examination, the patient was of normal build with a body mass index within the reference range. General physical and systemic examinations were unremarkable, and no craniofacial or airway abnormalities were noted on ENT assessment. Neurological examination revealed no focal deficits.

PSG was performed, which demonstrated SOREMPs with markedly short sleep latency of less than one minute, a feature highly suggestive of narcolepsy. Increased stage N2 sleep was observed, with multiple spontaneous arousals, though wake after sleep onset remained within normal limits. Snoring was noticed during the second half of the study, but there was no evidence of sleep-disordered breathing, apneic episodes, or nocturnal desaturation. The study also revealed periodic limb movements with a significant index greater than 15 per hour.

A subsequent MSLT was carried out following the PSG. The patient underwent four naps at two-hour intervals; a fifth nap was not performed once the diagnostic criteria were fulfilled. The mean sleep latency across naps was 1.25 minutes, confirming severe objective hypersomnolence. Moreover, SOREMPs were observed in three out of four naps, consistent with abnormal REM regulation (Table 1).

**Table 1:** Multiple Sleep Latency Test (MSLT) results showing nap-wise total sleep time, sleep latency, REM latency, and occurrence of sleep-onset REM periods (SOREMPs). The patient demonstrated a mean sleep latency of 1.25 minutes and three out of four SOREMPs, consistent with pathological hypersomnolence. A mean sleep latency of less than eight minutes with two or more SOREMPs is consistent with narcolepsy per the ICSD-3 criteria. REM: rapid eye movement; SOREMP: sleep-onset REM period; ICSD-3: International Classification of Sleep Disorders, Third Edition.

Nap	Total sleep time (min)	Sleep latency (min)	REM latency (min)	SOREMPs
1	28.0	2.0	-	No
2	19.0	1.0	1.0	Yes
3	18.5	1.5	1.5	Yes
4	19.5	0.5	0.5	Yes
Mean	21.2	1.25	0.65	3/4

**Figure 1:**
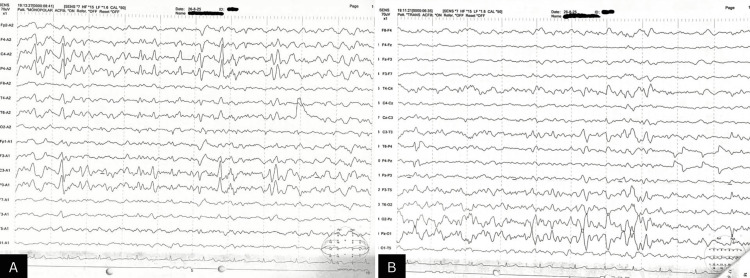
EEG of the patient demonstrating bilaterally symmetrical 30-50 μV sharp 8-9 Hz alpha activity over the central head region with transient lapses in awareness, consistent with absence seizures. (A) Monopolar referential montage demonstrating intermittent sharp wave discharges over the bilateral central region (C3 & C4) electrodes with preserved background activity, suggestive of epileptiform activity. (B) Transverse bipolar montage confirming the same ictal pattern with posterior predominance in the O2-Pz and Pz-O1 channels.

Given the family history of seizures, an EEG was performed, which revealed persistent bilaterally symmetrical 30-50 microvolt sharp 8-9 Hz alpha activity seen over the central head region with transient lapses in awareness, suggestive of absence seizures (Figure 1). This finding raised the possibility of epilepsy coexisting with narcolepsy. To further rule out organic or structural causes of secondary narcolepsy, a brain MRI was planned, although not yet performed at the time of reporting.

Based on the constellation of clinical symptoms, ESS score, sleep diary, PSG, and MSLT findings, along with EEG abnormalities, the patient was diagnosed with NT2 coexisting with absence epilepsy.

## Discussion

Under the ICSD-3 framework, narcolepsy is divided into two phenotypically distinct subtypes based on the presence or absence of cataplexy and hypocretin deficiency: NT1, defined by EDS with cataplexy and typically associated with low cerebrospinal fluid hypocretin-1, and NT2, in which EDS occurs without cataplexy and hypocretin levels are preserved [9].

Our patient fulfilled the diagnostic criteria for NT2, presenting with persistent EDS, REM-related phenomena, including hypnopompic hallucinations and sleep paralysis, absence of cataplexy, and objective confirmation of short sleep latency with two or more SOREMPs on the MSLT [9,16].

The diagnosis of narcolepsy in the pediatric and adolescent population is often delayed due to the attribution of symptoms to lifestyle, psychiatric illness, or behavioral problems. Registry and clinic-based studies have consistently documented prolonged diagnostic latency in narcolepsy, with many patients waiting close to a decade from symptom onset before receiving an accurate diagnosis [10,11]. In this case, the presence of mild snoring and a history of childhood OSA initially confounded the clinical picture, as residual OSA is a common cause of hypersomnolence. However, PSG excluded significant sleep-disordered breathing, highlighting the importance of objective sleep testing in the evaluation of EDS [9,16].

As per the AASM guidelines, the diagnostic protocol for narcolepsy requires PSG conducted under controlled laboratory conditions, followed the next morning by an MSLT to assess objective daytime sleep propensity [12,16]. In our patient, PSG showed a markedly short sleep latency of less than one minute and SOREMPs during the nocturnal study, with periodic limb movements but without apneic episodes or oxygen desaturation. MSLT revealed a mean sleep latency of 1.25 minutes with three SOREMPs out of four naps, meeting ICSD-3 criteria for narcolepsy [12,16]. These findings strongly supported the diagnosis, ruling out insufficient sleep syndrome and untreated OSA.

The patient experienced hypnopompic hallucinations and two episodes of sleep paralysis, both well-recognized REM-related phenomena in narcolepsy [9,12]. She did not report cataplexy, which distinguishes NT2 from NT1. Our patient’s episodes of incoherent examination writing exemplify automatic behavior, a semiautomatic continuation of ongoing activity during a microsleep, which has been documented in a substantial proportion of narcolepsy patients in published series [12,13].

The family history of seizure disorder raised a differential of epilepsy. However, the absence of ictal events, normal neurological examination, and the presence of REM-related symptoms with abnormal MSLT findings supported narcolepsy rather than epilepsy [14,17,18]. Psychiatric disorders such as depression and attention deficit hyperactivity disorder may also mimic EDS but lack objective PSG/MSLT correlates [9,12,16].

The heightened prevalence of periodic limb movements among narcolepsy patients observed in published studies may indicate common pathophysiological pathways governing sleep continuity, though the precise relationship remains to be elucidated [13].

This case emphasizes the diagnostic complexity of narcolepsy in adolescents, particularly in those with a history of OSA or overlapping symptoms. The functional consequences of undiagnosed narcolepsy are far-reaching: academic underperformance, impaired social development, and hazardous lapses of alertness during safety-critical activities represent avoidable harms that timely diagnosis can mitigate. Early diagnosis and initiation of therapy, encompassing pharmacotherapy (agents targeting wakefulness promotion and REM suppression) alongside structured behavioral strategies such as scheduled napping and sleep hygiene optimization, are crucial in improving outcomes [9,12,16].

## Conclusions

This case illustrates that NT2 in adolescents demands a high index of clinical suspicion, particularly when the diagnostic picture is complicated by prior sleep disorders, mood symptoms, or co-occurring epilepsy. Objective sleep laboratory evaluation remains indispensable in arriving at the correct diagnosis. Prompt identification enables targeted treatment and mitigates the substantial educational and psychosocial toll this disorder imposes on young patients, reinforcing the value of comprehensive neurologic and sleep assessment in this population.

## Figures and Tables

**Figure 2:**
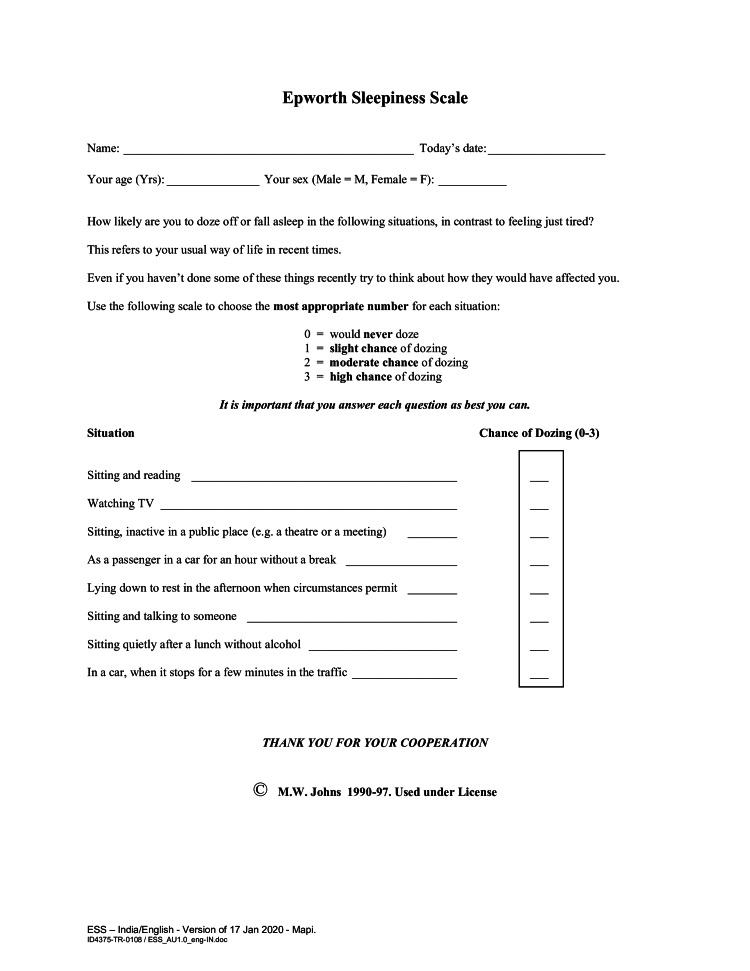
The Epworth Sleepiness Scale (ESS) is a validated, self-administered eight-item questionnaire developed by Murray W. Johns for the subjective measurement of daytime somnolence. Each item is rated on a 0-3 scale (0 = would never doze; 3 = high chance of dozing), yielding a maximum total score of 24. A score of 10 or above is considered indicative of excessive daytime sleepiness. In this case, the patient scored 19/24, reflecting severe subjective hypersomnolence. The ESS is protected by copyright (© M.W. Johns 1990-97) and is reproduced with permission obtained via the Mapi Research Trust (https://eprovide.mapi-trust.org/instruments/epworth-sleepiness-scale).

## Appendices

The Epworth Sleepiness Scale (ESS) is a validated, self-administered questionnaire developed by Murray W. Johns and is protected by copyright (© Murray W. Johns 1990-1997). Use in this publication was conducted with permission obtained via the Mapi Research Trust.
